# Elite Capture and Corruption in two Villages in Bengkulu Province, Sumatra

**DOI:** 10.1007/s10745-016-9837-6

**Published:** 2016-06-16

**Authors:** Anton Lucas

**Affiliations:** Flinders University, Adelaide, Australia

**Keywords:** Corruption, Elite capture, Local government, Conservation and development interventions, Sumatra

## Abstract

This paper examines leadership, elite capture and corruption in two villages in Sumatra. It compares implementation and outcomes of several conservation and development projects in the context of democratization and decentralization reforms introduced in Indonesia since 1998. In examining aspects of elite control and elite capture, this paper focuses on the activities of local elites, particularly village officials, who use their positions to monopolize planning and management of projects that were explicitly intended to incorporate participatory and accountability features. While elites’ use of authority and influence to benefit personally from their roles clearly reflects elite capture, there are nonetheless members of elite groups in these case studies who use their control of projects to broad community benefit. In both villages there is considerable friction between villagers and elites as well as among members of the local elite themselves over control of local resources. Differences in the structure of these cross-cutting internal relationships and of ties between local authorities and outside government and non-government agents largely explain the differences in degree of elite capture and its outcomes in the two cases.

## Introduction

This article examines conservation and development projects in two villages, Ladang Palembang and Bandar Agung, in the Lebong district of Sumatra’s Bengkulu province with a focus on leadership, elite capture and corruption. The article compares how government and non-government organization (NGO) funded conservation and development projects were managed during the decade following implementation of Indonesia’s 1999 decentralization legislation. In examining aspects of elite control and elite capture, this paper considers primarily the roles of village officials and secondarily that of external actors including district level government authorities, national park and forestry department officials, and NGO organizers, summarized in Tables 1 and 2.

**Table 1:** Socioeconomic characteristics, resources and stakeholders of conservation and development projects in Ladang Palembang village

Socioeconomic characteristics	Resource base	Conservation projects	Development projects	Stakeholders’ involvement				
				Village officials	Sub-district/ district officials	National park/Forestry Dept officials	NGOs	Villagers
Viillage comprises: • Three poor upland hamlets, inhabited by Rejang speaking descendants of original ethnic occupants, where dry agriculture is organized around women’s cultivating groups • Three more prosperous lowland hamlets inhabited by descendants of West Javanese transmigrants, primarily depend on wet-rice cultivation and gold mining. All village officials come from lowland hamlets	• upland hamlets: dry gardens and forest product collection •lowland hamlets: wet rice and gold mining	• Village regulations (Perda 2004a; Perda 2009) created three Village Protected Forests, and regulate use of local springs • Tree planting in buffer zones	PNPM • water system for lowland hamlet households • solar panels for upland hamlet households • PNPM incorporated participatory and accountability processes Women given special focus	•Administration of government programs; • Collaboration with WARSI • Headman extracted 5 % kickback from PNPM project	• Officials support both conservation and development projects. • Collude in some rent-seeking	• National park designation opposed by village, which requested to manage their own Village Protected Forests instead • Forest department poorly enforces regulations against logging and other illegal forest use	• Important influence of WARSI / Ford Foundation CBFM program in establishing and monitoring protected forests • Encouragement of women to play active management roles • no local journalists reporting corruption	• Women-run upland cultivation groups and monitor river fisheries • Lowland households, gardens and rice fields often run by women while men are away working in gold mines

**Table 2:** **Socioeconomic characteristics, resources and stakeholders of conservation and development projects in Bandar Agung village**

Socio-economic characteristics	Resource base	Conservation projects	Development projects	Stakeholders				
				Village officials	Sub-district/ district officials	National Park/ Forestry Dept officials	NGOs	Villagers
•Isolated village with small population living in dispersed hamlets with weak governance	• Some slash and burn shifting cultivation in the National Park buffer zone, but mainly sedentary agriculture outside the zone • wet rice fields owned by village elite • Illegal timber cutting, river fishing	• Rubber seedlings for reafforestation from Department of Agriculture	PNPM infrastructure projects: • new road, bridge, public bathing facilities, irrigation channels • housing • cattle project • rice for the poor • solar panels	•Village head monopolizes management of all projects; & 20 % kickback from PNPM project budgets & cuts from staff salaries •Village officials get priority in project benefits (cattle, housing) •Village development forum not functioning	• No meaningful oversight over dev’t projects • Collusion with village head, whose term of office was extended by district head	• Park headquarters located in another province on north-eastern side of park • no monitoring at either national or district level • Forestry department officials visit village rarely	• No NGO presence in the village, because headman is ambivalent towards NGOs • Journalists never visit, despite provision for this under PNPM program	•Disaffected and apathetic • Resent-ment towards village head because of corruption, cronyism and nepotism

Following Dasgupta and Beard (2007), this paper distinguishes between ‘elite control’ over project decisions that ensure access to project benefits by non-elites, and ‘elite capture’ of project benefits, which refers to “the process by which [local elites] dominate and corrupt community-level planning and governance” (Dasgupta and Beard 2007: 244, note 1)^1^. While the use of positions of influence to benefit materially from development projects is usually treated negatively as elite capture, there are members of these village elite groups who use their control over projects to benefit the wider community. It is argued here that the considerable degree of disaffection among ordinary villagers and friction between different groups among village elites concerning control of resources in these two cases give good reason to consider the conditions which promote the positive engagement of elite roles, as discussed in the Warren and Visser (2016) introduction to this themed issue.

In the Sumatran cases elite capture included examples of channelling development project benefits directly to members of the village elite (mainly the village head and other officials), demanding kickbacks from project budgets, and extracting illegal payments from community members in order to access benefits designed to alleviate poverty. Nonetheless, elite control of projects was not always associated with corruption, but in some circumstances lead to positive outcomes in which there was substantial community-wide benefit.

This article will also examine how beneficial elite control has operated in the implementation of community forest and water conservation projects (funded by the Ford Foundation through its local Sumatran NGO counterpart WARSI) in Ladang Palembang village, and less effectively in several village infrastructure development projects funded through the Indonesian government’s PNPM (National Community Empowerment Program) in both villages. These projects have checks and balances built into planning and implementation processes that are supposed to foster local empowerment and prevent corruption (McCarthy et al. 2014: 238, Fig. 10.1).

Elite control and elite capture have to be understood in the context of what one member of the village elite described as the “intertwining circles of leadership, family and money” so evident in Bandar Agung village (interview with AR, 8.04.2011). Because of the extraordinary stranglehold of particular political patronage relationships in Bandar Agung, both members of the elite who were outside the group controlling project funds and non-elite community members felt themselves powerless to affect the abuse of authority in Bandar Agung. The same disaffected member of the elite put it like this: “When we talk about justice we can only talk about applying the rules of government more fairly. But how can we do this if we are not members of the [village] government?” (idem).

Elite capture has been facilitated by decentralization policies in Indonesia which gave local elites increased access to resources, and paradoxically by the role of the political party system that has emerged since the end of authoritarian rule in 1998, although this has been more evident in studies at district than at village level (Heryanto and Hadiz 2005; Aspinal and Mietzner 2010; Van Klinken 2012)^2^. In some cases it is possible to track the direct connection between elite capture of resources through official positions and corruption. The cuts or kickbacks taken from externally funded project budgets, (which amount to 5 to 20 % in the two villages) can clearly be defined as corruption. They occurred because of formal capture of the management of these projects by the village head in each case. Elite capture and corruption of external and government funded conservation and development projects were found to have occurred more frequently during the second term of office of the headmen of both villages in this study. Villagers explained this increase in corrupt behavior as a reflection of the need to accumulate wealth and pay off political debts while they were still in office and had opportunities to do so.

**Fig. 1:**
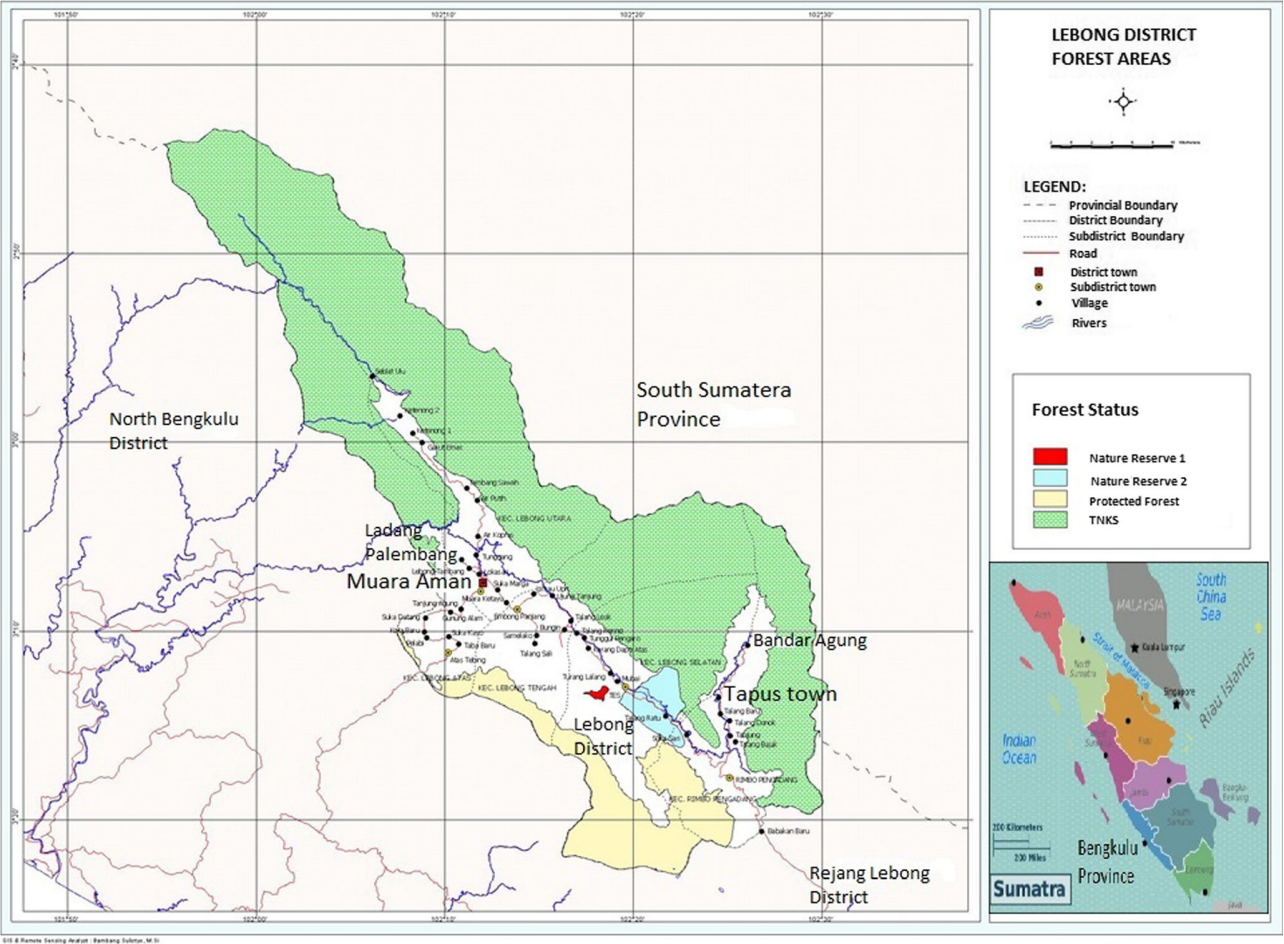
Map of Lebong District (Bengkulu Province) research villages and forest areas

Both villages are located in Lebong district, which borders on Kerinci Selabat National Park (TNKS) created in 1996 (see map, Fig. 1). Villagers still burn the forest for new gardens inside park buffer zones, while the park itself is a source of valuable forest products for both communities^3^. Despite criticism of their manipulation of development funds, the headmen of both villages were seen to be supportive of conservation campaigns to protect forest remnants. In Ladang Palembang an *adat* (customary law) based Village Regulation established rules for Village Protected Forests (Perda 2004a, re-issued as Perda 2009), and in Bandar Agung a customary (*adat*) ceremony to preserve the balance of nature and provide ritual security against occasional sightings of Sumatran tigers around the village was revived (Sastro 2006). Actual enforcement of laws on exploitation of forest products from the national park was a more complicated matter.

The sections that follow trace the roles of the two village heads and other agents in the implementation of conservation and development initiatives in the villages of Ladang Palembang and Bandar Agung, Sumatra. Research in the two villages was conducted over several months during 2010 and 2011 as part of a comparative research project on social capital and natural resource management. A survey of forty households, selected by geographically stratified random sampling, was carried out in each of the two Sumatran villages. Survey questions covered basic demographic and socio-economic information for each household, as well as probing attitudes to local government and natural resource management issues, and assessing levels of participation in decision-making and conservation and development program interventions (see Tables 1 and 2). In-depth interviews were subsequently carried out with key informants representing the range of social groups and leadership positions in each community^4^.

## NGO Engagement and Protected Forests in Ladang Palembang Village

Findings from the research project survey (*n* = 40) carried out in Ladang Palembang village in early 2010, showed that most respondents valued WARSI’s conservation and social action program. WARSI (the Indonesian Conservation Community), a local NGO, worked in partnership with the Ford Foundation’s Community Based Forestry Management (CBFM) program on social development and conservation in four Sumatran provinces^5^. A key focus of these programs in Ladang Palembang was the creation of three protected forest areas, and a water supply project for the village sourced from one of the protected springs. A village regulation was written with WARSI’s assistance for that purpose (Perda 2004a).

According to WARSI’s former regional director, Ladang Palembang village was chosen because of the need to resolve the conflict over a new transmigrant settlement in the upland areas of the village affecting the buffer zone of the national park in 2000–2001. Supported by the Bengkulu Peasants’ Union (StaB), indigenous hamlets there were reluctant to give up their land (Bachriadi 2011). WARSI also campaigned successfully to have the licenses of three sawmills in the buffer zone cancelled, effectively aborting the transmigrant settlement^6^. Most of the old growth forest outside the national park had been logged in the mid-1980s by a private company clearing land for a rubber plantation. Alongside protecting important watershed forests overlapping with the national park boundaries, WARSI also wanted to implement a community development program to improve village livelihoods adjacent to the park buffer zone by providing productive rubber seedlings and candlenut (*Aleurites molucana*) trees. The Ladang Palembang village head was involved in both projects:

Before I had not given any thought to conservation. Then WARSI asked us: “Why don’t you make a village [protected] forest and an *adat* [protected] forest?” That was in 2002. We got together the well-known community leaders^7^ from the village to talk about how to protect nature, particularly our springs. The local leaders agreed that if the forest were cleared we would lose our springs. We heard a story about how a village head had stopped an illegal logger – presumably using persuasion^8^. This was an example we needed to follow. We did a survey of our forests involving women, youth, farmers, village officials and the village council [BPD]. We went and put numbered metal plates on trees to mark the village protected forest boundaries. WARSI helped us with GPS mapping. When we had finished the mapping, we invited our *adat* people to make a regulation. (Interview with village head in Ladang Palembang, 7.03.2010)

After the boundary mapping, a village regulation was drafted with the participation of various stakeholders including WARSI, village officials, the newly elected village council (BPD) and several women’s groups, to protect the three blocks of village protected forest (Perda 2004a)^9^. The regulation specified customary *adat* fines for any unauthorized cutting of trees or new garden plots in these forests that were to be reported to the village head^10^. The new village regulation also set out permitted uses of the protected forests, including collecting fruits, medicinal plants and honey “without destroying or cutting down trees, but ensuring their protection.” Farmers with gardens bordering the village protection forests who burned off ferns and *alang-alang* grass (*Imperata cylindrica*) are required to build a two-meter wide ditch to prevent the fire from spreading. They also had to re-plant critically degraded areas (Perda 2004a, §2).

The same village regulation applied to gardens remaining inside the national park and village protected forest after boundaries were drawn. Although technically illegal under national regulations, maintaining gardens inside the national forest was a local compromise whereby these pre-existing gardens were tolerated, but could not be enlarged, nor new ones created. Cultivators were required to plant timber species and to protect the forest. The cultivators could keep their rights to forest products (such as wild honey and forest fruits), but they did not have the right to cut down the trees that produced these products. Article 7 (Perda 2004a) stipulated that cutting down old or unproductive trees required permission and that replacement trees had to be six months old. Gardens located in so-called regeneration areas could remain, and any cutting of trees could only take place with permission of the local Farmer’s Conservation Group which was set up in Ladang Palembang village as part of WARSI’s conservation program.

The village regulation governed water use as well as land. Article 6 specifically banned the taking of fish from the Ketaun River using illegal methods such as poison, electric shock, and dynamite. Sastro (2003a, 2003b) reports that women cultivators were involved in evicting fish poachers using these illegal methods on several occasions during this period, indicating active local support for these environmental protection measures partially based on Rejang customary law. The fact that women cultivators were the main arm of enforcement was due to the importance of their local community cultivation groups, again based on Rejang adat^11^. The village regulation on natural resources stipulated various sanctions if the above rules were broken. Apart from the customary fines mentioned above, there were also fines based on the price of the timber that was cut, damaged or burnt. Gardens illegally opened in the village protected forest had to be restored to their original boundaries. Heavy fines were stipulated for killing fish using any illegal means^12^.

How successful was the application of these sanctions in preventing illegal cultivation and illegal logging within these newly designated village protected forests? And what was the role of village leadership and village elites in this process? In fact, the attempt by WARSI to re-establish Rejang customary law (*adat*) penalties to protect the village’s natural resources was never seriously enforced by the official village head or the village customary leadership after the WARSI programme ended. What it did do was raise the awareness of the importance of conservation amongst farmers. WARSI’s attempt to give a revived role to an *Adat* Council to hear cases of breaches of natural resource management rules was also intended to raise awareness of conservation issues (Tim Fasilitasi CBFM 2003). In theory, according to the village regulation, both the village head and local officials have to examine each case (Perda 2004a). If there was proof of a violation of forest protection regulations, the matter was handed over to the *Adat* Council, comprising Rejang elders, village officials and Islamic leaders, which convened a public meeting to hear the case^13^.

In the only recorded case heard by the village *Adat* Council, the WARSI-initiated Farmers’ Conservation Group had reported a local upland farmer named Rahman for cutting down two trees (three cubic meters of timber). Rahman confessed, explaining that his economic situation was difficult, his wife was sick, and he was two months in arrears with unpaid school fees. A witness confirmed his situation. An accomplice admitted showing Rahman the location of the trees and introducing him to the Chinese middleman who bought the logs, but denied any knowledge of where they had been cut. The *Adat* Council chairperson, although acknowledging that the accused was motivated by economic hardship, gave his advice based on the wider significance of the environmental protection issues concerned in the case:

The area where the act occurred is a source of springs; the steep terrain is prone to landslides, it is the home of rare animals, etc., and if the environment is damaged, it will destroy the balance of nature that will be a disaster for many people. (Minutes of the *Adat* Council 2002)

After hearing a plea for a light sentence because of the personal circumstances of the accused, and the fact that he admitted his wrongdoing, the *Adat* Council imposed fines in kind and directed Rahman to donate the value of the timber he had cut to the village to be used to rehabilitate the village protection forest.

The example shows how WARSI promoted the integration of customary, Islamic and state law and succeeded in raising awareness of the importance of conservation in the community, particularly the connection between forest protection and water security. The *Adat* Council regulations specified that while existing gardens within the village protection forest could still be cultivated, they could only be planted with originally wild but now cultivated perennial rainforest trees like rubber, durian, jackfruit, or a bean called *petai* (*Parkia speciosia*). Cutting trees for timber or clearing land by slash and burn techniques were not allowed. As noted above, this customary (*adat*) rule was written into official village regulations, which protected the existing cultivation rights of the community but at the same time prevented the opening up of new gardens within the protection forest. Hamlets near the park were asked to report any illegal cutting of timber, the person doing it, and the position on the protection forest’s boundary (Ladang Palembang village head, 7.3.2010).

WARSI’s knowledge-sharing approach had several results in Ladang Palembang and nearby villages. One was the broad realization that forests were needed to protect water sources, and that the village could impose *adat* penalties on people cutting timber in village protected forest areas, with or without district-level endorsement. WARSI’s programs consciously engaged both the elite and non-elite members of the community. The WARSI sponsored forest conservation program of measuring and marking the Village Protected Forest boundaries emphasized the link between protecting forests and water sources. This knowledge sharing, formalized in the writing of the village regulations and in the settlement of village boundary disputes, helped protect the national park (TNKS) as well as local water sources. WARSI was behind the cancellation of sawmill permits and supported protest actions by women cultivators against illegal fishing.

WARSI support for poor village women in enforcing regulations against damaging fishing practices that threatened their livelihood indicates the complex equity challenges facing conservation interventions. Strict conservation measures disproportionately affected poor upland forest-dependent Rejang hamlets more than they did the more prosperous lowland West Javanese hamlets. On the other hand, protection of riverine resources was important to the upland subsistence base. As part of their advocacy for natural resource management, the presence of WARSI undoubtedly helped empower upland women to act against outside poachers using damaging fishing methods, and suggests the wider influence and potential empowerment through conservation interventions that meet up with local needs and management practices. The *Adat* Council’s effectiveness was probably connected to WARSI’s presence in the village; after the project ended, there are no records of further meetings.

## Elite Capture and Environmental Regulation in Bandar Agung Village

State law prohibits logging and other forest product collection in national parks. However, locals regularly enter the national park to collect forest products for which there are ready markets, and villagers never report illegal collection of forest products by other villagers. Illegal exploitation is driven by poverty and market demand (Ribot and Peluso 2005), and is maintained by cultivating collusive relations with local authorities responsible for reporting and enforcement.

Sapwood or eaglewood (*gaharu*) is a globally highly valued forest product used to make perfumes and incense, hence locally much sought after because of its high price. But its collection requires cutting out the sapwood that is infected by a parasitic mould. Exported to China and Thailand for the cosmetic industry, the tree has to be cut down to obtain the sapwood, which is sold in the district town of Muara Aman to middlemen for between IDR 5–15 million (USD 416–1250) per kg.

The headman of Bandar Agung acknowledged that the collection of all forest products was illegal in protected national parks. But he claimed to be both unable and unwilling to stop it. He described his attitude to enforcement when a group of outside collectors from the Lebong district market – three hours away – stopped at his house on their way to the national park to search for this increasingly scarce and highly profitable sapwood:

Giving them warnings will only make things difficult for us. They’ll still go into the forest. So it’s better if we don’t know. Then it means they go there at their own risk. Even if we have a responsibility to tell them [its wrong], they will still do damage to the forest. So it’s better that we don‘t know about it … The village headman is a leader, not a law enforcement officer. (Interview with Bandar Agung village head, 28.03.2011)

The village head claimed that admonishing the sapwood collectors would not work. The price is a big incentive for those determined to exploit the increasingly scarce trees, and they have no other source of livelihood as profitable. Instead of bypassing his house to get to the Park, better that they enter and leave along the track below his house.

I feel bad about what they are doing. But there is no easy way out. We want a positive outcome. They know that what they are doing is breaking the law. We can warn them, but when they are determined to go there, there’s no point. It’s better to just let them go. If I warn them they will tell their friends “If you go to the village head’s house you’ll get a warning”. Next time they go into the Park, they will use the track along the river to avoid meeting me. So nothing is achieved. These five men have brought provisions to live in the park for two weeks. [If] they don’t report what they are doing to us, then we aren’t responsible, and we don’t have to warn them or report them [to the police]. (Interview with Bandar Agung village head, 28.03.2011)

Indeed state law enforcement can be problematic in Indonesian villages. The police in Indonesia are based at subdistrict level and do not have any direct village level representation. The village head would have to bring the sapwood offenders to the police office in the subdistrict centre of Tapus (see Fig. 1) in order for them to be charged with illegal activities. Tapus is one hour’s drive away on an unsealed road which becomes impassable and dangerous after heavy rains. He would have to provide proof of identity and a witness. And he would need to bring to the police the confiscated sapwood as evidence that they were stealing forest products.

The problem of enforcing conservation regulations is an issue throughout Indonesia’s vast, but rapidly degrading, forest estate. Park boundaries are long and porous. The Kerinci Selabat National Park borders 4 neighboring provinces and 13 districts. With a total boundary of 2638 km, park authorities have a huge task (Taman Nasional Kerinci Sebelat 2007; see map, Fig. 1). Bandar Agung village has park boundaries on three sides. Park rangers located at the TNKS headquarters on the opposite northeast side of the park in faraway Jambi province do not have an office in Lebong District. Consequently, coordinated actions against taking protected species by the Park’s authorities in surrounding villages are often impractical.

In poor regions such as this, village officials and even department authorities sometimes ignore infractions out of sympathy with the situation of poor rural people. Two Bandar Agung villagers, who make a livelihood from selling live songbirds caught in the National Park are an example. They were from two of the poorest families in the village, and the headman of Bandar Agung made no attempt to stop them (Lucas 2011). In return for looking the other way, authorities would receive illegal payments from those whose exploitation of park resources were unhindered. Although it is not clear whether this was the case with the Bandar Agung villagers who engaged in songbird collecting, undoubtedly the Bandar Agung village head was receiving kickbacks from the outside collectors of the lucrative *gaharu* sapwood. Otherwise there would have been no reason for him to entertain visits from these illegal collectors, and ignore (or encourage) their discrete use of the walking track below his house while refusing to report their activities. Overseeing illegal forest product collection was completely consistent with his blatant extractions from development projects within the village, facilitated also by collusive patronage relationships he was able to maintain with higher-level authorities in the District and Forest Department offices.

## Elite Control of a Water Supply Development Project in Ladang Palembang

Until recently very few villages in Indonesia had access to a domestic water system (known locally as PAM Desa)^14^ and most relied on wells or streams. During the WARSI project period (2001–2004) there were discussions about the problem of local springs made muddy during the wet season and drying up in the dry season. A domestic water supply piped to all six hamlets in the village from five different springs was planned as one of the WARSI projects (Perda 2004b). The 2004 village regulation then was extended to become the basis for a National Community Empowerment Program (PNPM) financed village water supply scheme in 2008. The scheme involved construction of a holding dam at the spring, a 2 km underground pipeline to the village, with water hydrants to each group of 4–6 households connecting running water to 80 % (92) of the 114 lowland households. Each household is charged a fee of IDR 5000 per month, regardless of the amount of water used. The project was built and run by the village head’s two brothers-in-law, who are credited with providing transparency in the construction of the project, as well as accountability and technical management in the subsequent running of the scheme, for which there were no regulations in PNPM guidelines.

The planning of the project went through the required PNPM participatory procedures. However, because of their isolation from the centre of village power, and the location of the spring to be used, the three upland Rejang hamlets were not included in the PNPM stage of the water supply project. This was due partly to the PNPM consultation process, which villagers in the three upland hamlets felt had left them out. The members of these upland hamlets claim they were not invited to meetings, while the PNPM village facilitator said that when a meeting was held in an upland hamlet, there were too few attending to make a quorum, leading the inexperienced young facilitator to (wrongly) conclude that they “did not care about PNPM”^15^.

Ladang Palembang village is divided between two main social groups: the descendants of pre-war colonial transmigrants from West Java who live in three lowland hamlets, and the local Rejang people inhabiting the three upland hamlets who cultivate subsistence dryland crops and practice occasional slash and burn agriculture. The upland Rejang people live in scattered settlements, speak the Rejang language, have their own prayer houses, and only go to the village centre to take children to school or collect monthly rice rations. Lowland views on the Rejang uplanders are usually negative. Village officials are all members of the lowland pre-war transmigrant elite. The marginality of the upland hamlets in Ladang Palembang was a significant issue in both the NGO sponsored conservation and government PNPM development programs. Sponsoring the revival of the village *Adat* Council through the WARSI intervention attempted to bridge the gap between the two ethnic groups, but, as mentioned earlier, no further meetings were recorded after WARSI left the village in 2006. The subsequent failure of the PNPM project facilitator to press for their inclusion in the water supply development program, reflects what upland hamlet dwellers have long felt as an ongoing disregard for their welfare by lowland village officials. On the other hand, inclusion of lowland women in externally facilitated conservation and development programs seems to have established important new participatory practices. The solar panel program for upland hamlets (not a PNPM project) can be seen as compensation by the village head for the upland hamlets’ exclusion from PNPM water supply project benefits.

With respect to financial management, unlike PNPM projects in Bandar Agung village to be discussed below, transparency mechanisms, including broad-based distribution of responsibilities and multiple checks and balances during construction of the Ladang Palembang water system, were relatively effective and prevented officials making money from its construction. During the construction stage the project manager refused to pay a 5 % kickback to the village headman, his brother-in-law, although this was to have repercussions the following year. He also made every effort, in line with PNPM principles, to employ women as much as possible during the construction phase^16^.

The sustainability of a village water supply system requires planning, transparency and accountability not only in construction, but also in the ongoing collection and management of water fees, and in remuneration for those who maintain the system. Round the clock maintenance work was carried out by AS (another brother-in-law of the village head, from the same elite family that had held the office of village head for many decades), with a husband and wife team, also from among lowland elite families, collecting and recording water payments.

The village water system is run by a committee of three members - me, D, and her husband, H. We hold monthly meetings at the village office. Only half of the 92 families that have connected to the system usually attend. The agenda including what kinds of mutual help (*gotong royong*) for maintenance will be discussed. The secretary, D [who also performs the role of treasurer at the request of the women who wanted a woman to look after the money], reports on income and expenditure. If we want to change the water fees, the regulation has to be changed which means a new meeting. In May 2009 a meeting was held attended by 41 subscribers. I got selected by lottery. (Interview with AS in Ladang Palembang, 14.03.2010)^17^

The PNPM village water system started operations in May 2009, with 88 subscribers; by March 2010 there were 91, of which 14 (12 %) were behind in their payments by one or two months. The rule is that if they are in arrears more than three months their access to water will be disconnected^18^.

Village water supply systems are high maintenance projects. The Ladang Palembang scheme was an example of beneficial elite control, with one of the headman’s brothers-in-law (AH) building the project and the other brother-in-law (AS) managing it. The latter maintained the pipes and hydrants for a monthly wage of IDR 150,000 (USD 12.50)^19^. There was a separate budget of IDR 100,000 (USD 8.30) for parts. The treasurer of the water supply system of Ladang Palembang village kept the register of connected households, which showed who was behind with monthly water payments, and she managed the maintenance budget together with AS. The treasurer herself was a member of the economic elite and reputedly owned a hectare of irrigated rice fields. As in the case of Ladang Palembang’s Village Protected Forests, the village regulation on water was important to what lowland villagers saw as a fair management system^20^. For example, in March 2010 there were jokes about the village secretary’s unpaid water fees – he was two months in arrears. According to the water regulation, if fees remained unpaid after three months, water supply would be disconnected. Asked how water payments were collected from village officials, the water system treasurer said:

When we collect water payments, the village secretary, who hasn’t paid since January, is treated in the same way as everyone else. After three months of non-payment their water is cut off, regardless of who it is or what their position is. Our job is what’s important. Because there was no regulation before, the village officials could do what they liked. Now that we have the village regulations as the mandate for action, we’re not afraid to ask people to pay, whoever they are. The village secretary is a village leader, and he’s setting a bad example. It’s as if he is telling people “It’s OK not to pay”, or he’s telling them how to get into a debt trap. (Interview with secretary of Ladang Palembang village water scheme, 15.3. 2010)

The conflict between the village head and his two brothers-in-law over control of the PNPM water system mentioned earlier shows that ‘the’ village elite is not a politically consolidated entity and that intra-elite rivalry may contribute to more open processes and better governance. The power play between the headman and AH, his older brother-in-law who ran the PNPM water system project in the village, illustrates this:

When we had the first [PNPM] village planning meeting for 2009, I was going to continue as leader of the work group. But the village head didn’t attend the meeting. I was told later that he went to the PNPM subdistrict office, saying: “If AH becomes the leader of the work group [again] I won’t sign off on the village planning meeting.” The subdistrict facilitator asked me what was going on in the village as the headmen had come to the subdistrict office, and complained that I was “too inflexible”. At the second PNPM village meeting while the village head was speaking I cut him off^21^ and told the meeting that I was resigning, although I was older than him and he had no power over me. When the PNPM Team came from Jakarta they told me that the headman hadn’t signed off on my project report, because the village stamp had “gone missing”. (Interview with HK, Ladang Palembang PNPM work group leader, 29.03.2010)

The Ladang Palembang village head then appointed another (younger) brother-in-law as leader of the project work group who gave him the kickback of 5 % on all materials for the PNPM village laneways project (Interview with former work group leader in Ladang Palembang, 7.04. 2010). A WARSI activist had this to say about the performance of the Ladang Palembang head in the village election of 2007:

The village head is a rather closed person, not transparent as far as financial reporting is concerned. He will grab money from a project if he doesn’t have any. And he chooses who has access to projects. It’s not an open process, but is controlled by himself and two of his wealthy supporters. But when he has money he is generous with people who need it. (Pers. com. with ex-WARSI organizer, 05.03.2010)

## Corruption and Elite Capture in Bandar Agung Development Projects

While the situation in Ladang Palembang village development projects exhibits both ‘elite control’ with significant benefits to the wider population and some ‘capture’ on the part of the village head, in Bandar Agung village elite control has become synonymous with systematic capture and corruption. This can take various forms. Firstly, the village head demands illegal payments from villagers to qualify for benefits from provincial and district projects that are meant to be free. Public grants and benefits for which Bandar Agung villagers were forced to make a cash payment to the headman included provision of gas cookers (the compulsory contribution was IDR 30,000 [USD 2.50]); a solar panels program (IDR 150,000 [USD 12.50)^22^; and monthly allocations of 15 kg rice under the ‘rice for the poor’ program (IDR 15000 [USD 1.25]). In Bandar Agung, the rice for the poor distribution is conducted on the front veranda of the village head’s house. According to villagers, 175 kg of undistributed rice was later sold off, while two of the poorest families in the village (the songbird-catcher families mentioned above) were refused rice because they could not afford the cash payment demanded.

The village head can extort these illegal payments because, as official administrative village chief, he has free reign with project implementation in the village in the absence of meaningful oversight on the part of district level authorities, or through internal village administrative accountabilities (McCarthy et al. 2014: 255–256). The original decentralization legislation (Law 22/ 1999) had provided for checks and balances through an independently elected village council (BPD). With the rationale that this had lead to conflict between executive and legislative arms, the revised regional government Law 32/ 2004 reversed this by replacing elected councils with appointed consultation boards with restricted authority. The Indonesian government’s community empowerment program, PNPM, was intended to have a broadly based management system, beyond executive control, but in varying degrees it proved not to be immune to entrenched forms of elite capture.

Outright elite capture also occurred when the village head and a small circle of his supporters directly appropriated community development project benefits. For example, cattle were distributed to those with family ties, then supporters and cronies. It is widely remarked that those who receive such project benefits are ‘KSK’ (*Kelop Sama Kades*), ‘in cahoots with the headman’. When Bandar Agung village received 18 cows as part of a government cattle rearing project, the headman reputedly took 8 animals for himself. The remaining cows were distributed among village officials and relatives (Interview with former Bandar Agung village official, 16.4.2011). The headman illegally determined the distribution of project benefits as well as work group membership for the government’s PNPM community empowerment program according to this ‘KSK’ principle. In Bandar Agung out of 20 new houses built for the first phase of a new village transmigrant housing scheme, 8 were allocated to the Bandar Agung headman’s family and to village officials who were the headman’s cronies. Not all officials are involved with these illicit arrangements, but even those who resist, stay quiet because their positions are on the line.

Why is there no resistance from within the village or demand for accountability from any superior levels of government to date? The Bandar Agung village head had manipulated the 2001 village elections by stacking the village election committee with his own supporters, so he could alter the village electoral roll, allowing non-residents who cultivated plots of land in the village to vote. (He allegedly threatened that he would burn down their field huts if they did not vote for him.) He also got the village election committee to agree that household heads could vote on behalf of other household members eligible to vote, which is illegal. Reputedly, some of the ‘original’ villagers set fire to his house in protest over the election result. These incidents have had a lasting effect on village governance, as no one is prepared to openly oppose the village head, despite regarding him as having “no moral authority”. (Interviews in Bandar Agung village, March 2011).

The Bandar Agung village head also introduced the practice of taking a cut from all village officials’ salaries during his first term of office. At the end of every quarter he would go to the subdistrict office and ‘collect’ the wages of village staff without their authority:

He knew from the subdistrict treasurer the date we were going to be paid, and he went off by himself and collected our wages. When he got back here we should have received IDR 450,000 but he took IDR 150,000 for ‘transport expenses’. When we complained he derided us saying: ‘You don’t work, but you are paid a salary just to sleep’. (Interview with ES, a former Bandar Agung village official, 03.04.2011)

This practice would not be difficult to remedy, villagers say, if the wages of village officials were paid directly into bank accounts. But in a remote village like Bandar Agung even salaried officers find it inconvenient to store money in a distant bank. The kickback culture between village and district offices is strongly embedded across the country, where elected officials condone it in return for support for their political re-election campaigns.

We have already described an example of ‘benevolent’ elite control (Fritzen 2007: 1360) in the case of the water supply development project in Ladang Palembang, where demands for kickbacks faced resistance from the village head’s own family members, and which for the main part was implemented and managed in the public interest by other elite families. In contrast, in Bandar Agung, corruption pervaded all of its development projects. Clearly, such blatant misappropriation and nepotism could not have continued without the collusion of higher levels of government. The example of the PNPM infrastructure project, including irrigation channels for rice cultivation, illustrates the tangle of collusive arrangements that made village level elite capture on this scale possible.

In 2007 Bandar Agung was given funding for ten PNPM Infrastructure work groups (LKD) to build a bathing and toilet facility and irrigation channels for the rice fields. The village head called the work group leaders together and told them he was going to manage nine of these groups himself for a 20 % ‘fee’. Also, he told them that he would return the funds budgeted (IDR 15 million) for one ‘fake’ infrastructure project that was not going to be implemented, to the PNPM subdistrict managers as a kickback. “This will ensure we get another project next year,” he said^23^. The village head forged signatures on quarterly work group reports^24^. The 20 % fee meant that projects ran out of money or had to be finished with below standard materials, while the stipulated 10 % management fee for LKD work group members was never paid. In March 2010, the nine work groups were told that they would be amalgamated under one group with the total budget to be cut by 20 %. As a result the road was shortened from 700 m to 500 m but this still left a budget shortfall. The headman told the meeting that this had to be made up by “voluntary contributions to costs from each work group” (Notebook of LKD member copied on 19.04.2011)^25^.

The renovation of the suspension bridge over the Ketaun River was another notorious case of project gouging. In 2011, repairs to the bridge and renovated irrigation channels were financed from the same PNPM Infrastructure program. The village head accompanied the work groups to the bank in Lebong district town to collect the project funds. When they returned to the village he gave them a choice. Either hand over the project money to him to manage or, if they wanted to run the project by themselves, to pay him a kickback of 20 %. PNPM management rules require that work groups manage their own project expenditures, including contracts for construction materials.

## How Does Elite Capture Work?

In the undoubtedly extreme case of Bandar Agung village, elite capture is characterized by two main issues: collusion with higher levels of government and the inadequacy of civil society organization. At district level there was a complete lack of project monitoring and enforcement of regulations. A former village official commented: “I said to the [Bandar Agung] village head that what he was doing [managing work group project funds himself] was against PNPM rules…. PNPM work groups were supposed to manage project finances and hire contractors themselves. His response was: ‘Who cares what the PNPM rules are?’” Project materials were not brought from district-level wholesale suppliers according to the cheapest quotes, but through favored supporters or clients of the village head. Collusion with district officials who share the same political party interests fuels the systematic nation-wide dimensions of local corruption. Lebong district was no exception to this as the close relationship between village and district heads was widely known. The latter extended the former’s first term of office, and visited Bandar Agung village during the headman’s election campaign for a second term. The Bupati was also re-elected for a second term.

An inadequate foundation for institutional checks and balances within the village governance framework, in particular since 2004, by the unelected and disempowered Village ‘Consultation’ Board (BPD), is another key part of the elite capture scenario. Villagers’ responses to the incidents discussed show that they regarded themselves as powerless to oppose the Bandar Agung village head. Those leaders sensitive to public opinion sometimes had to pay a financial price for maintaining their reputations. Because of the illicit appropriation of project funds, on two occasions PNPM infrastructure project leaders in Bandar Agung had to use personal funds to enable projects to be completed. The suspension bridge renovations were short of IDR 8 million from a 30 million rupiah budget because of kickbacks.

Apart from the important structural issues just mentioned, it is worth noting that the village head of Bandar Agung is locally known as both a clever speaker and a hospitable person. But his friendly manners conceal resentments and grudges against people who are opposed to his misappropriations, manipulation of work groups and favoritism towards relatives and supporters who ‘agree’ with him. Because of these attitudes, villagers hold long-term rancour and feel disconnected from village governance (Interviews in Bandar Agung, April 2011). From his point of view, the thoroughly reprobate Bandar Agung village head complained, “People don’t appreciate what I have done, they are apathetic and never come to meetings.” (Interview in Bandar Agung, 12.03.2011). However, village meetings are few and far between. Only four meetings were listed on the village notice board for the period December 2009 – April 2010; three of these were scheduled outside the village.

Is the situation regarding leadership and elite capture in Bandar Agung an extreme case? Apparently so. When work group members from the village met with work groups from other villages in Lebong district, while collecting PNPM project payment instalments from the bank in Muara Aman, they found out that other village heads in the subdistrict did not directly manage work group finances; rather they provided supervision and coordination only under limited circumstances as provided by PNPM guidelines. However, because of the extraordinary stranglehold of the network of corrupt payments integrated into the personal and political system the village head has created in Bandar Agung, there is nothing in the short term they could do with this information. Reporting the problem to the subdistrict PNPM officials would not achieve anything, as they too were receiving kickbacks from the village head. Regional journalists seldom come to remote villages like Bandar Agung to report on village level corruption because they prefer to cover governance issues at district level, and because village level corruption is regarded as relatively small compared to national cases, hence not considered newsworthy (Bengkulu journalist, pers .com., 29.10.2013).

That said, there is growing evidence that some of the mechanisms for dealing with local level corruption are taking effect. Several village heads in Lebong district have been charged with corruption of PNPM funds in recent times (WARSI activist, pers. com., 18.10.2014). A national corruption investigation into PNPM found that from 2004 to 2013, IDR 250 billion (USD 20.8 million] has been misappropriated by groups receiving money through the program. Attempts to use litigation have recovered IDR 2.2 billion (USD 183,300] while IDR 33.3 billion (USD 2.77 million) was “in the process of being recovered”. In 2013 there were 22 cases of misuse of PNPM funds in projects amounting to more than IDR 1 billion (USD 83.3 thousand). According to a national PNPM bureaucrat and poverty reduction official in the Coordinating Ministry for Public Welfare, these figures are small compared with the annual PNPM budget of IDR 10–11 trillion (USD 833–916 million), or a total of IDR 56 trillion (USD 4.66 billion) of project expenditures so far^26^.

## Policy Recommendations

The causes of elite capture are primarily due to the failures of higher-level government oversight and the weakness of countervailing village institutions to balance the power of village elites. We have noted that the move towards village democratization began with the establishment of elected village representative councils (BPD) under Law 22/1999 (Antlov 2002: 362–375). The process of expanding village council participation in decision-making was reversed with the 2004 legislative revision, which reinstated a more or less monolithic executive authority at the village level. If participation in village governance is to increase, the first order of priority is to strengthen democratization through formal and independent election of hamlet heads and of village councils, whose legislative parity with the village head must also be restored. The new 2014 Village Law 6/ 2014 does provide for election of BPD members, but does not yet clearly give the council equivalent authority to the village head, and does not address the role of constituent hamlets at which level most direct public participation traditionally took place.

Secondly, there is the issue of transparency in both villages. The fact that none of the village regulations are publicly available, for example on the village notice boards or in the village office, needs addressing. Village officials say they do not have copies, that they have been lent to other people, or were never circulated. Because of WARSI’s involvement in making the water regulation of Ladang Palembang village, there were copies in this NGO’s archive. However, in Bandar Agung village with no NGO program comparable to WARSI, records could not be found. Recently the Minister for Village Affairs, Underdeveloped Regions and Transmigration announced he was sending a letter to all villages requiring that project expenditure acquittals be posted on village notice boards, “so people would know how the money was spent”^27^.

Thirdly, the role of the media in information dissemination, particularly about corruption below the village level, needs to be maintained, Falsifying work group reports recently emerged as another form of corruption within PNPM projects, according to a Bengkulu-based journalist who says that local corruption of PNPM Infrastructure projects throughout the province has been known for some time. Journalists and NGO-based facilitators are meant to provide further check and balance mechanisms, but sometimes also engage in collusion. “From information that I have obtained, a number of rogue [PNPM] facilitators have ‘taught’ local civil society groups who are implementing PNPM programs how to corrupt funds. It is not uncommon for facilitators themselves to ask the work groups (LKD) for money to write their final reports for them, when in fact these reports have to be written by the work groups themselves” (Bengkulu journalist, pers. com. 6.11.2013)^28^.

Fourthly, there is a need to strengthen NGO engagement at village level and institutionalize more internal participatory community monitoring mechanisms to increase accountability of officials at the village level as well as of external authorities at the subdistrict and district levels. In the neighboring province of West Sumatra, independent monitoring of PNPM projects led to the development of a provincial monitoring system to be implemented by NGOs “so that communities have safe channels to frankly express their opinions, suggestions and complaints” (Indrizal 2008; LASP 2007). Unfortunately, no agreement has yet been reached on national standards or systems for independent project monitoring by NGOs in Indonesia (Indrizal, pers. com., 3.12.2015). This process was started at the national level by the Institute for Professional Certification of Community Empowerment Facilitators (LSP-FPM) during former President Yudhoyono’s last term of office, but became caught up in a tug of war over authority and responsibility between the Ministry of Home Affairs (which administered PNPM) and the new Ministry of Village Affairs, Underdevelopment and Transmigration (Kemendes), which under current President Jakowi, has different policy concerns (creating ASEAN sponsored village economic communities through direct grants for village enterprises). The community development and empowerment funding previously processed through the PNPM program has now been incorporated in modified form into the Village Law of 2014, substantially increasing the power of the village head.

## Conclusion

This paper provides insights into how elite control and elite capture operate at village level and how internal relations of rivalry and collusion are affected by external interventions and political arrangements. Particularly in regard to development projects, which typically involve access to substantial resources, village heads in both study villages attempted to tightly control access and distribution of benefits. Because of the less concentrated organization of power amongst the primary elite family in Ladang Palembang village, however, the extent of capture by the village head was constrained. The PNPM water project leader refused to give the village head (his brother-in-law) the kickbacks demanded, while the running of the water system was managed with generally acknowledged transparency by another brother-in-law, known as an opponent of the headman. In this case elite control, in Fritzen’s sense of benevolent capture (Fritzen 2007: 1360), generally worked positively to achieve benefits for non-elite villagers.

By contrast in Bandar Agung development infrastructure projects failed to meet participation and accountability objectives because of the monopoly of power in the hands of the village head and collusion with higher authorities. All villagers knew about the corruption, but those who were not part of the Bandar Agung village inner circle, had to be very careful about what they said and to whom. Importantly, these examples of corruption and elite capture of the PNPM infrastructure projects in Bandar Agung village were unchallenged by overarching government institutions and have been characterized by an absence of any meaningful participation in village monitoring, despite community ‘capacity building’ and ‘empowerment’ being key objectives of this national community development intervention. Notwithstanding PNPM program efforts to ensure accountability, the village head ruthlessly shored up his position using nepotism, cronyism and information control.

Especially after the revised local government law (UU 32/ 2004) reduced the independent monitoring capacity of village councils, village institutional structures facilitated elite control and capture with power once more concentrated in the role of village head, resulting in a situation where officials have a strong sense of entitlement to moneymaking activities. This takes place in the context of a political party system in which elected district and provincial heads and assembly representatives have made money politics an all-pervasive aspect of the processes of democratization itself (McDonald 2014: 203). While at the same time an independent anti-corruption agency (KPK) attempts to tackle overt high-level corruption, its systemic underpinnings remain unaddressed.

Both villages border the buffer zone of the Kerinci Selabat National Park. In regard to conservation, the presence of an outside NGO in the village of Ladang Palembang promoted broader public participation in decision-making. WARSI used village regulations as a means of empowering communities to protect forests and water resources, and to manage projects that benefited the community. WARSI expanded the traditional roles of women as heads of cultivation groups. Women participated in writing agreements on natural resources and in managing village water system finances. They also became village officials and council members. Incorporation of customary practices involving the minority Rejang uplanders and dominant West Javanese lowlanders have brought some, though uneven, benefits to the indigenous upland minority.

While Ladang Palembang has village regulations (not always enforced) to protect its remaining threatened forest and springs, there is still ample supply of water and timber in Bandar Agung. The village head there claims that he wants to protect the forest, and has urged the villagers to plant trees through informal agreement with cultivators, although they did not see the need to do so, because “there is plenty of timber in the national park” (Interview with Bandar Agung villager, 10.4.2011). It is said the village head has let selected logging trucks into village forest areas in the past but on a much smaller scale than officials at the district level^29^. His attitude to poaching in the national park hardly supports his pro-conservation claims.

Two national level institutional shifts will potentially increase the importance of village level governance for natural resource management and community development. A recent Constitutional Court ruling (2012) determining that customary *adat* forest does not come under the state forest regime will place greater authority over forest resources in local hands (Rachman and Siscawati 2014)^30^. And the new Village Law (UU 6/ 2014), transferring community development funding directly to village government, makes the importance of checks and balances in local governance regimes a core issue for forest conservation as well as for improving local livelihoods sustainably. Leadership, public participation and mechanisms to ensure accountability will be even more critical to these outcomes throughout Indonesia.

## Footnotes

1 Dutta (2009) considers that elite capture occurs when there is a bias in the selection of project beneficiaries, such that only some groups benefit.

2 The national PNPM community development program intentionally bypassed district-level government in making payments directly to village level work groups for community projects that were meant to be determined at village meetings. See McCarthy et al. (2014) for details of the complex processes that were aimed at local transparency and accountability.

3 468 villages across four provinces share borders with the TNKS buffer zone. In Lebong district, 48 villages are located along the borders of the Park, which takes up 59 % of the district's area (113,512 ha) (See Figure 1).

4 All interviews referred to in this paper were recorded in Indonesian and Rejang languages and later translated into English by the author, with the assistance of Nurkholis Sastro for Rejang language interviews.

5 For a brief history see http://kkiWARSI.wordpress.com/WARSI-itu/.

6 Without permanent housing or land, all but a few transmigrant families had left the area by the end of 2003.

7 That is "people who are respected and who have followers in the village because of their honesty, care and involvement in the community" (Sastro, pers. com., 1.04. 2011).

8 WARSI’s approach was to use persuasion; the environmental action group WALHI used advocacy of conflict cases through the media and the courts (Sastro, pers. com., 23.04.2011).

9 The national park administration had not responded to the village’s proposal to create new areas of protected village forest at the time fieldwork ended. However, local people have generally respected these commitments regardless of lack of upper level government recognition.

10 The Rejang adat penalties used by the village government for illegally cutting trees was a 2–3 meter roll of white cloth, 16 kg of rice, and the conduct of a community adat ritual meal to restore harmony in the village. The official village regulation stipulated a penalty of two tins of rice (article 9), which amounts to 32 kg. Also, the illegal logger had to reimburse the village for the value of the illegally cut timber.

11 Ladang Palembang village has four such cultivation groups involving women from settlement communities of 4–6 dwellings, often little more than huts, located throughout village uplands. Led by local Rejang women, they cultivate gardens, collect firewood and edible forest plants, and catch fish in the Ketaun river. With WARSI support, they reacted strongly to livelihood threats from outside poachers (Sastro 2003a: 28).

12 If the illegal fishermen used violence resulting in death, the fine was one house, equivalent to IDR 8 million rupiah (USD 660). If animals were killed they had to be replaced. If fish were killed the fine was the value of one quarter of a house, or 2 million rupiah (Perda 2004a : clause 13).

13 The Adat Council chair was an elder of the lowland hamlets, reflecting their control of formal village institutions.

14 Since PNPM began, some 16,000 villages in Indonesia have built domestic water systems run by local communities (G. Acciaoli, pers. com., 7.07.2014).

15 Interview with the village PNPM project facilitator in Ladang Palembang, 6.3.2010. On these lowland and upland communities, see Prodolliet and Heinzpeter (1992), and Schneider (1995).

16 Active participation of women in decision-making was prescribed in the PNPM program (McCarthy *et al*. 2014:251). Earlier, WARSI had also encouraged women's involvement in both conservation and village governance roles including drafting the village regulation (WARSI 2005; Sastro 2003a; b).

17 Under the local lottery system, names of those who are interested in a position are written down on pieces of paper, and then lots are drawn. It wasn’t an appointed position, but not exactly elected either.

18 There was an initial connection fee of IDR 10,000 (USD 0.83) to the water system. The management committee set monthly water rates (payments) at IDR 5000 (USD 0.40). Monthly water payments in a large household in the district capital of Muara Aman at this time were IDR 20,000 (Sastro, pers. com., 2.05.2015).

19 The Bengkulu regional minimum wage in 2010 was IDR 780,000 per month (USD 65).

20 Upland villagers also needed a water system but were left out for reasons discussed above.

21 Cutting off the village head while he was speaking at a public meeting even by someone with the same or higher status was unheard of, and indicated how angry HK was.

22 At this time USD 1.00 = IDR 12,000.

23 Interview with a PNPM work group member who attended this meeting, 19.04.2011. The communal toilet block remained permanently locked and could not be used because no toilets were ever built inside them (Interview with a Bandar Agung hamlet head, 12.03.2011).

24 When shown the work group LKD quarterly report, the work group leader said: "These 16 signatures are fake” (Interview in Bandar Agung, 19.04.2011).

25 Reportedly, corruption of an earlier PNPM Infrastructure project in Bandar Agung (2009) caused that project bridge to collapse after heavy monsoonal rains because the bridge had not been built to specifications.

26 ‘Ratusan Miliar dana PNPM Mandiri di Korupsi’, *Tempo*, 8 March 2014. I am grateful to a local Bengkulu based journalist for this information.

27 ‘Penyaluran hampir tuntas: penggunaan diawasi ketat’ *Kompas* 27.12.2015.

28 In North Bengkulu district, a PNPM facilitator charged IDR 3 million (USD 250) to write a report for a village PNPM work group leader. In his defence the work group leader said he did not know how to write a project report, and it would probably have cost him the same amount to go to the subdistrict, find the documents and write the report himself. 'Terima Beres, Bayar Rp.3 Juta', *Rakyat Bengkulu* 13.01. 2014.The local press needs village level reporters to expose this corruption, although with the gradual closing of district level newspapers, it is becoming more difficult for the media to expose this kind of corruption.

29 Illegal logging is widespread in Lebong district, where an estimated 70 % of timber used for district development construction is processed from illegally logged timber most of it coming from protected forest in conservation areas. In 2007 the head of the local district forestry office reported a member of the Lebong district assembly (DPRD) for his involvement in illegal logging. One year later the district head’s adjutant who was coordinator of the Lebong District Illegal Logging Control Team, was himself caught by police while transporting illegally logged timber to the site of the Bupati’s new official residence then under construction (Lucas and Bachriadi 2009).

30 On the legal and political ramifications for *Adat* communities and the Forestry Department to implement this ruling see Rachman and Siscawati (forthcoming).

## References

[CR1] ‘Ratusan Miliar Dana PNPM Mandiri di Korupsi’.(2014) Tempo 8 March 2014.

[CR2] ‘Terima Beres, Bayar Rp.3 Juta’,(2014) Rakyat Bengkulu, 13 January, 2014.

[CR3] Antlov H (2002). Negara dalam Desa: Patronase Kepimpinan Lokal.

[CR4] Aspinal E, Mietzner M (2010). Problems of democratisation in Indonesia: elections, institutions and society.

[CR5] Bachriadi, D. (2011). Between Discourse and Action: Agrarian Reform and Rural Social Movements in Indonesia Post-1965. PhD thesis, Flinders University at http://theses.flinders.edu.au/public/adt-SFU20110222.150002/index.html

[CR6] Dasgupta A, Beard VA (2007). Community driven development, collective action and elite capture in Indonesia. Development and Change.

[CR7] Dutta, D. (2009). Elite Capture and Corruption: Concepts and Definitions, a Bibliography with an Overview of the Suggested Literature. NACER (National Council of Applied Economic Research).

[CR8] Fritzen SA (2007). Can the Design of Community-Driven Development Reduce the risk of elite capture? Evidence from Indonesia. World Development.

[CR9] Heryanto A, Hadiz V (2005). Dilemmas of democratic consolidation in Indonesia. The Pacific Review.

[CR10] Indrizal, E. (2008). Pendampingan Pemantauan Independen PNPM Berbasis Masyarakat Desa. Province Based Monitoring-Community Participatory Monitoring (PBM-CPM) PNPM Mandiri Pedesaan: Sebuah Pengalaman Pembelajaran di Propinsi Sumatra Barat. LASP (Lembaga Analisis Sosial dan Pembangunan (Institute for Social and Development Analysis) presentation to PNPM National Meeting, Jakarta 28 April–-1 May.

[CR11] LASP (Institute for Social and Development Analysis), (2007). Kerangka Acuan Kerja (TOR) Kegiatan Pemantauan Independen di Tingkat Propinsi (Province-Based Monitoring)

[CR12] Lucas, A. (2011). Catching songbirds in a National Park, Inside Indonesia 106, Oct–Dec http://www.insideindonesia.org/feature-editions/catching-songbirds-in-a-national-park

[CR13] Lucas, A. and Bachriadi, D. (2009). Trees, Money, Livelihood and Power: the Political Ecology of Conservation in Bengkulu Province in the Era of Decentralisation. Paper to the International Conference: Legal Pluralist Perspectives on Development and Cultural Diversity, Zurich: 31 August–3 September

[CR14] McCarthy J. F. et al.(2014). Dilemmas of participation in the National Community Empowerment Program. In Hill H. (ed.), Regional dynamics in a decentralised Indonesia, ISEAS, Singapore, pp. 233–259.

[CR15] McDonald H (2014). Demokrasi: Indonesia in the twenty-first century.

[CR16] Minutes of Adat Council Session (2002). Pembicaraan dalam Peradilan adat.

[CR17] Perda 2/2004a. Peraturan Desa Ladang Palembang Kecamatan Lebong Utara Kabupaten Lebong Nomor 02 Tahun 2004 Tentang Hutan Lindung, Hutan Adat dan Kawasan Penghijauan Desa Ladang Palembang

[CR18] Perda 5/2004b. Peraturan Desa Ladang Palembang Kecamatan Lebong Utara Kabupaten Lebong Nomor 05 Tahun 2004 Tentang Pengelolaan Sumberdaya Air Desa Ladang Palembang.

[CR19] Perda 5/2009. Peraturan Desa Ladang Palembang Kecamatan Lebong Utara Nomor 05 Tahun 2009 Tentang Hutang Lindung Desa, Hutang Adat Desa dan Kawasan Penghijauan Desa Ladang Palembang

[CR20] Prodolliet S, Heinzpeter Z, King VT (1992). Illusory worlds and economic realities: the gold of Lebong. The Rejang of southern Sumatra.

[CR21] Rachman NF, Siscawati M (2014). Masyarakat Hukum adat Adalah Penyandang Hak, Subjek Hukum, Dan Pemilik Wilayah Adatnya: Memahami Secara Konteksual Putusan Mahkamah Konstitusi Republik Indonesia Atas Pekara Nomor 35/PUU-X/2012.

[CR22] Rachman, N. F and Siscawati, M. (forthcoming). ‘Forestry Law, Masyarakat Adat, and Struggles for Inclusive Citizenship in Indonesia’, in Christoph Antons (ed) *Routledge Handbook of Asian Law*, London and New York: Routledge.

[CR23] Ribot J, Peluso N (2005). A theory of access. Rural Sociology.

[CR24] Sastro N. (2003a). Perempuan dalam Lingkaran Politik Desa dan Sumber Daya air: Studi Kasus Perempuan Ladang Palembang. Alam Sumatera.: 26–28.

[CR25] Sastro N. (2003b). Rumpian Ala Perempuan Ladang Palembang. Alam Sumatera: 28–29.

[CR26] Sastro N (2006). Tuntutan Alam Setelah Dua Tahun Tidak Kedurai: Pengalaman Warga Bandar Agung-Tapus. Petalangan.

[CR27] Schneider J (1995). From upland to irrigated Rice; the development of wet-Rice agriculture in Rejang Musi, Southwest Sumatra.

[CR28] Tim Fasilitasi CBFM Kabupaten Rejang Lebong, 2003. Meletakkan Posisi Tawar dan Komitmen Bersama Melalui Kesepakatan Adat Rejang. Laporan Tahun 2003 Jaringan Daerah WARSI Bengkulu.

[CR29] TNKS [Taman Nasional Kerinci Sebelat] (2007). Situasi dan Kondisi Taman Nasional Kerinci Seblat Seksi Konservasi Wilayah II Bengkulu [unpublished document].

[CR30] UU 22/1999 Undang Undang Tentang Pemerintah Daerah (Regional Government Law] no 22, 1999

[CR31] UU 32/2004 Undang Undang Pemerintah Daerah [(Revised) Regional Government Law] no 32, 2004

[CR32] UU 6/2014 Undang Undang Desa [Village Law] No. 6, 2014

[CR33] Van Klinken, G. (2012). Countries at the crossroads 2012: Indonesia. Freedom House Series, at https://freedomhouse.org/report/countries-crossroads/2012/indonesia

[CR34] Warren C, Visser L (2016). The Local Turn: An Introductory Essay on Leadership, Elite Capture and Good Governance in Indonesian Conservation and Development Programs. Human Ecology.

[CR35] WARSI (Komunitas Konservasi Indonesia) (2004). ‘Laporan Akhir: program Ikhtiar Sosial Menuju Terwujudnya Desentralisasi Pengelolaan Sumberdaya Alam Berbasikan masyarakat di Sumatra Bagian Selatan’ (Periode Oktober 2000 – June 2004). Kerjasama antara The Ford Foundation dengan Komunitas Konservasi Indonesia (WARSI)

